# Patterns of Telemedicine Use and Glycemic Outcomes of Endocrinology Care for Patients With Type 2 Diabetes

**DOI:** 10.1001/jamanetworkopen.2023.46305

**Published:** 2023-12-06

**Authors:** Margaret F. Zupa, Varsha G. Vimalananda, Scott D. Rothenberger, Jonathan Y. Lin, Jason M. Ng, Rozalina G. McCoy, Ann-Marie Rosland

**Affiliations:** 1 Division of Endocrinology and Metabolism, University of Pittsburgh School of Medicine, Pennsylvania; 2 Center for Health Outcomes Research, Veterans Affairs Bedford Healthcare System, Bedford, Massachusetts; 3 Division of General Internal Medicine, University of Pittsburgh School of Medicine, Pennsylvania; 4 Division of Endocrinology, Diabetes, and Nutrition, University of Maryland School of Medicine, Baltimore; 5 University of Maryland Institute for Health Computing, Bethesda; 6 Center for Health Equity Research and Promotion, Veterans Affairs Pittsburgh Healthcare System, Pennsylvania; 7 Department of Medicine, Boston University School of Medicine, Massachusetts

## Abstract

**Importance:**

Telemedicine can increase access to endocrinology care for people with type 2 diabetes (T2D), but patterns of use and outcomes of telemedicine specialty care for adults with T2D beyond initial uptake in 2020 are not known.

**Objective:**

To evaluate patterns of telemedicine use and their association with glycemic control among adults with varying clinical complexity receiving endocrinology care for T2D.

**Design, Setting, and Participants:**

Retrospective cohort study in a single large integrated US health system. Participants were adults who had a telemedicine endocrinology visit for T2D from May to October 2020. Data were analyzed from June 2022 to October 2023.

**Exposure:**

Patients were followed up through May 2022 and assigned to telemedicine-only, in-person, or mixed care (both telemedicine and in-person) cohorts according to visit modality.

**Main Outcomes and Measures:**

Multivariable regression models were used to estimate hemoglobin A_1c_ (HbA_1c_) change at 12 months within each cohort and the association of factors indicating clinical complexity (insulin regimen and cardiovascular and psychological comorbidities) with HbA_1c_ change across cohorts. Subgroup analysis was performed for patients with baseline HbA_1c_ of 8% or higher.

**Results:**

Of 11 498 potentially eligible patients, 3778 were included in the final cohort (81 Asian participants [2%], 300 Black participants [8%], and 3332 White participants [88%]); 1182 used telemedicine only (mean [SD] age 57.4 [12.9] years; 743 female participants [63%]), 1049 used in-person care (mean [SD] age 63.0 [12.2] years; 577 female participants [55%]), and 1547 used mixed care (mean [SD] age 60.7 [12.5] years; 881 female participants [57%]). Among telemedicine-only patients, there was no significant change in adjusted HbA_1c_ at 12 months (−0.06%; 95% CI, −0.26% to 0.14%; *P* = .55) while in-person and mixed cohorts had improvements of 0.37% (95% CI, 0.15% to 0.59%; *P* < .001) and 0.22% (95% CI, 0.07% to 0.38%; *P* = .004), respectively. Patients with a baseline HbA_1c_ of 8% or higher had a similar pattern of glycemic outcomes. For patients prescribed multiple daily injections vs no insulin, the 12-month estimated change in HbA_1c_ was 0.25% higher (95% CI, 0.02% to 0.47%; *P* = .03) for telemedicine vs in-person care. Comorbidities were not associated with HbA_1c_ change in any cohort.

**Conclusions and Relevance:**

In this cohort study of adults with T2D receiving endocrinology care, patients using telemedicine alone had inferior glycemic outcomes compared with patients who used in-person or mixed care. Additional strategies may be needed to support adults with T2D who rely on telemedicine alone to access endocrinology care, especially for those with complex treatment or elevated HbA_1c_.

## Key Points

**Question:**

What is the association between telemedicine use and glycemic outcomes among adults with varying clinical complexity receiving endocrinology care for type 2 diabetes from 2020 to 2022?

**Findings:**

In this cohort study including 3778 adults, there was no significant change in estimated hemoglobin A_1c_ (HbA_1c_) over 12 months (−0.06%) among patients using telemedicine alone, while patients who used in-person (−0.37%) and mixed care (−0.22%) had significant HbA_1c_ improvements.

**Meaning:**

These findings suggest that patients with type 2 diabetes who rely on telemedicine alone to access endocrinology care may require additional support to achieve glycemic goals.

## Teaser

This cohort study evaluates patterns of telemedicine use and their association with glycemic control among adults receiving endocrinology care for type 2 diabetes.

## Introduction

Use of telemedicine to deliver endocrinology care for type 2 diabetes (T2D) increased dramatically in 2020 and is likely to continue given a shortage of endocrinologists and persistent barriers to in-person visits.^1,2,3,4^ Prior randomized clinical trials have demonstrated the efficacy of telemedicine, defined as synchronous audio or video communication between patients and practitioners, for improving glycemic outcomes for adults with T2D.^5,6,7,8,9^ However, data are lacking on utilization patterns and outcomes of routine telemedicine care for T2D since 2020, especially in the endocrinology setting.^10,11,12,13^ Examination of clinical outcomes is critical, as patients with T2D may be more diverse, clinically complex, and face additional barriers to accessing care than clinical trial participants.^14,15^ Moreover, telemedicine interventions for diabetes that have been tested in clinical trials frequently include intensive care components that are not routinely implemented in current diabetes telemedicine care, such as remote monitoring of blood glucose, multidisciplinary team care, and patient engagement between visits.^5,16,17,18,19^

An understanding of which patients with T2D have continued to use telemedicine and how their glycemic outcomes vary across different clinical scenarios is necessary to identify which patients can successfully manage their diabetes with telemedicine alone and which may need additional support or in-person care to reach treatment goals. Endocrinology practitioners have expressed concern that patients with increased medical complexity may be less well served by telemedicine care.^19,20^ Although guidelines recommend use of telemedicine to increase access to diabetes care, additional evidence is needed on short- and long-term clinical outcomes across distinct patient populations to guide best practices.^21,22,23^ In this study, we aimed to address this evidence gap by evaluating (1) the characteristics of adults with T2D who persisted in using telemedicine-only vs those who switched to in-person or mixed endocrinology care after initial telemedicine use early in the COVID-19 pandemic, (2) the association of these care modalities with glycemic outcomes, and (3) how factors that contribute to clinical complexity, including insulin regimen and comorbidities, are associated with glycemic outcomes across these different care modalities.

## Methods

### Study Design and Clinical Setting

This retrospective cohort study included adults with T2D who were seen via telemedicine for either an initial or follow-up visit between May 1 and October 31, 2020, in the endocrinology division of a large health system, which includes more than 30 practitioners across 8 clinics in rural and urban counties. Similar to other care settings, most patients with T2D in this health system are managed by their primary care practitioners. However, patients may receive care from an endocrinologist through referral by a health care practitioner or self-referral, with associated costs varying according to their specific heath insurance plan. In this clinical setting, use of telemedicine vs in-person care was based on individual patient and practitioner preference and availability; there were no blanket policies determining visit modality. To reduce bias in assessment of ongoing telemedicine use and glycemic outcomes, we focused on patients with capability to use telemedicine at baseline. This study was approved by the University of Pittsburgh institutional review board and determined to be exempt from informed consent as it involves secondary research on data collected as part of routine care. The results are reported in accordance with Strengthening the Reporting of Observational Studies in Epidemiology (STROBE) reporting guidelines.^24^

### Study Cohort

**Figure 1.**
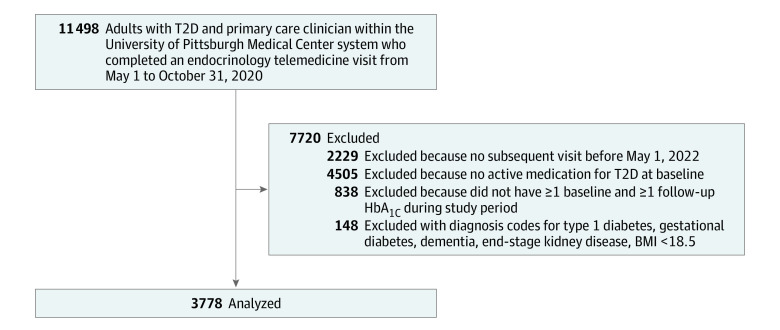
Flowchart of Study Cohort BMI indicates body mass index (calculated as weight in kilograms divided by height in meters squared); HbA_1c_, hemoglobin A_1c_; T2D, type 2 diabetes.

Patients were included if they had at least 2 hemoglobin A_1c_ (to convert from percentage to proportion of total hemoglobin, multiply by 0.01) values and 1 subsequent encounter in the division of endocrinology during the follow-up period, which extended through May 1, 2022. A diagnosis of T2D was based on encounter diagnosis codes (*International Statistical Classification of Diseases and Related Health Problems, Tenth Revision [ICD-10] *code E11.X). Patients had to be over age 18 years, have a primary care practitioner within the health system to ensure adequate capture of comorbidities, and at least 1 prescription for an antihyperglycemic medication at baseline, to exclude patients seen in endocrinology for another condition who also have T2D controlled without medication. Patients with type 1 diabetes, gestational diabetes, end-stage kidney disease, and dementia were excluded. Details on cohort creation and inclusion and exclusion criteria can be found in the cohort flowchart in Figure 1.

### Measures

All data were extracted from electronic medical records. Telemedicine encounters were defined as completed synchronous visits with an endocrinology practitioner including audio-visual or audio-only communication. Encounters designated as phone calls, which are used to provide unscheduled support between visits, were excluded. Baseline factors including patient demographics and clinical characteristics were extracted as close to the initial qualifying visit date as possible starting May 1, 2020. The first HbA_1c_ value recorded in the study period was defined as the baseline value; follow-up HbA_1c_ values were collected through May 1, 2022, and had to be at least 10 weeks apart. Body mass index (BMI) was categorized into standard levels as shown in Table 1. Comorbidities of interest included cardiovascular disease and psychological conditions documented at least twice in outpatient encounters during the study period (see eTable 1 in Supplement 1 for *ICD-10* codes). These were chosen to include 1 category of concordant comorbidities, which have management strategies similar to T2D (eg, coronary artery disease), and 1 category of discordant comorbidities with management unrelated to T2D (eg, bipolar disorder) to assess whether these had distinct associations with glycemic outcomes.^25,26^ Insulin prescription was based on active medication orders at baseline and categorized as no insulin, basal insulin only, or multiple daily injections (MDI; ie, both basal and prandial insulin). Social Deprivation Index (SDI), a composite measure of local area deprivation linked to health outcomes, was based on 5-digit zip code.^27,28^ Rural-urban commuting area (RUCA) codes were used to assess rurality, which also impacts diabetes care and outcomes.^29,30,31,32^ Race was extracted from the electronic medical record, based on patient self-report on clinical intake forms, and was included in this study to evaluate racial and ethnic variation in patterns of telemedicine use because race and ethnicity have previously been associated with factors, such as health insurance and physical environment, that affect both patterns of health care use and glycemic outcomes. Patients were separated into 3 categories: telemedicine only, in which all visits in the study period were conducted via telemedicine; in-person only, in which all visits after the initial telemedicine visit were in-person; or mixed follow-up, in which patients had both telemedicine and in-person visits following the initial visit in the study period.

**Table 1.** Baseline Characteristics of Patient Cohort

Characteristic	Participants, No. (%)				
	Total (N = 3778)	Telemedicine only (n = 1182)	In-person only (n = 1049)	Mixed follow-up (n = 1547)	*P* value^a^
Age, mean (SD), y	60.3 (12.7)	57.4 (12.9)	63.0 (12.2)	60.7 (12.5)	<.001
Sex					
Female	2201 (58)	743 (63)	577 (55)	881 (57)	<.001
Male	1577 (42)	439 (37)	472 (45)	666 (43)	
Race					
Asian	81 (2)	23 (2)	14 (1)	44 (3)	<.001
Black	300 (8)	114 (10)	77 (7)	109 (7)	
White	3332 (88)	1024 (87)	944 (90)	1364 (88)	
Other or missing^b^	65 (1)	21 (2)	14 (1)	30 (2)	
Hispanic or Latino	66 (2)	18 (2)	5 (0)	43 (3)	<.001
Not specified or missing	158 (4)	63 (5)	45 (4)	50 (3)	
SDI Score, mean (SD)	40.5 (23.9)	38.3 (25.3)	41.4 (22.0)	41.7 (23.9)	<.001
RUCA					
Urban	2645 (70)	926 (78)	669 (64)	1050 (68)	<.001
Suburban	836 (22)	204 (17)	260 (25)	372 (24)	
Rural	297 (8)	52 (4)	120 (11)	125 (8)	
HbA_1c_, mean (SD)%	7.6 (1.7)	7.6 (1.8)	7.4 (1.6)	7.7 (1.7)	<.001
Insulin					
No insulin	1476 (39)	528 (45)	435 (41)	513 (33)	<.001
Basal only	652 (17)	199 (17)	180 (17)	273 (18)	
MDI	1650 (44)	455 (38)	434 (41)	761 (49)	
No. of noninsulin medications, mean (SD)	1.9 (1.1)	1.8 (1.1)	1.9 (1.1)	1.9 (1.1)	.05
Body mass index^c^					
18.5-24.9	196 (5)	53 (4)	69 (7)	74 (5)	<.001
25-29.9	639 (17)	169 (14)	228 (22)	242 (16)	
30-34.9	880 (23)	211 (18)	291 (28)	378 (24)	
35-39.9	688 (18)	207 (18)	198 (19)	283 (18)	
>40	770 (20)	247 (21)	195 (19)	328 (21)	
Missing	605 (16)	295 (25)	68 (6)	242 (16)	
Comorbid conditions					
Cardiovascular	1393 (37)	401 (34)	418 (40)	574 (37)	.02
Psychological	1246 (33)	454 (38)	277 (26)	515 (33)	<.001
Appointments per 12 mo, mean (SD), No.	2.6 (1.0)	2.1 (0.8)	2.5 (1.1)	2.9 (0.9)	<.001
No. of follow-up HbA_1c_ test results per 12 mo, mean (SD)	1.7 (0.8)	1.4 (0.8)	1.8 (0.8)	1.8 (0.8)	<.001

Abbreviations: HbA_1c_, hemoglobin A_1c_; MDI, multiple daily injections; RUCA, rural-urban commuting area; SDI, social deprivation index.

SI conversion factor: to convert to proportion of total hemoglobin, multiply by 0.01.

^a^ For continuous variables, Kruskal-Wallis test was used; for categorical variables, χ^2^ test was used.

^b^ Other includes Indigenous American, Alaska Native, Samoan, Other Pacific Islanders, and those who indicated their race was unknown, not specified, declined to answer, or for whom race data was missing.

^c^ Body mass index is calculated as weight in kilograms divided by height in meters squared.

### Statistical Analysis

We summarized baseline patient characteristics using frequency (percentage) for categorical variables and mean (SD) or median (IQR) for continuous variables. Baseline characteristics were compared among those who used telemedicine only, in-person only follow-up, and mixed follow-up using χ^2^ tests for categorical variables and Kruskal-Wallis tests for continuous variables. To examine the outcomes of excluding patients without follow-up HbA_1c_, we also compared characteristics of excluded patients with the modeled cohort. Each demographic variable that differed between the modeled cohort and excluded patients was included as a covariate in final model.

Our primary outcome was 12-month HbA_1c_ change, with a secondary outcome of HbA_1c_ change at 24 months to explore longer-term glycemic outcomes. We used a linear mixed model fitted via maximum likelihood estimation to assess HbA_1c_ change by follow-up care modality and clinical factors. Random effects for patient and practitioner were included, with the endocrinology practitioner of the initial encounter serving as the unit of the random practitioner effect. Variables of main interest were treated as fixed effects and included follow-up care modality, insulin treatment regimen, and composite binary indicators for presence of cardiovascular disease or psychological conditions. To allow HbA_1c_ response to vary over the follow-up period, we included a quadratic function of time, defined as number of months since baseline HbA_1c_. To capture between-patient variability in trajectories of HbA_1c_ response, a random slope for time was also incorporated. We adjusted for patient age, sex, race, ethnicity, SDI, RUCA category (urban, suburban, rural), baseline HbA_1c_, and BMI (with patients with missing BMI included in a discrete level) to control for potential confounding. To quantify HbA_1c_ change over time within follow-up care modalities and test whether HbA_1c_ change differed significantly between modalities, we included 2-way interactions between follow-up care modality and fixed effects for time. Least-squares means (LS means) estimation quantified HbA_1c_ change over time within follow-up modalities, and contrasts of LS means estimated differences in HbA_1c_ change over time between modalities.

We conducted 2 additional analyses. First, a subgroup analysis limited to patients with baseline HbA_1c_ 8% or higher was performed to further explore outcomes specifically for patients with elevated HbA_1c_. Second, difference-in-difference analyses contrasted LS means estimates of HbA_1c_ change for patients with insulin use and comorbidities vs those without between care modalities and between patients who used telemedicine vs mixed or in-person care between levels of insulin use. Two- and 3-way interactions between time, care modality, and insulin regimen, as well as interactions between time, care modality, and comorbidities, were included in the mixed models. All analyses assumed a type 1 error rate of .05 and were performed using SAS software, version 9.4 (SAS Institute Inc). Data were analyzed from June 2022 to October 2023.

## Results

### Characteristics of Patients by Care Modality

There were 3778 patients in the final cohort, with a mean (SD) age of 60.3 (12.7) years, 58% female (2201 participants), 2% Asian (81 participants), 8% Black (300 participants), and 88% White (3332 participants) (Table 1). The plurality of patients (1547 participants [41%]) used mixed modalities after the initial telemedicine visit, with similar proportions of patients (1182 participants [31%]) using telemedicine only and in-person only (1049 participants [28%]) over the study period. Patients who used telemedicine only were younger and more likely to be women and Black than patients in the in-person and mixed follow-up groups. In addition, patients who used telemedicine only had less local area deprivation (SDI) and were more likely to be urban dwelling. The in-person follow-up cohort had lower baseline HbA_1c_ compared with the other cohorts. Patients in the telemedicine-only cohort were more likely to have a psychological comorbidity and not be prescribed insulin at baseline than patients in the other 2 cohorts.

Comparison of patients included in final models with those excluded due to lack of follow-up HbA_1c_ demonstrated significant differences in demographics (eTable 2 in Supplement 1). Excluded patients were more likely to be younger, women, Black, urban dwelling, not prescribed insulin, have lower baseline HbA_1c_, and have fewer visits per year than included patients.

### Patterns of Care Utilization

The proportion of endocrinology visits for T2D that were conducted via telemedicine was highest from May to October 2020 at 84% (10 987 of 13 031 visits), dropped to 63% from November 2020 to April 2021 (4923 of 7783 visits), then to 42% from May 2021 to October 2021 (3373 of 8053 visits), and stabilized at 41% from November 2021 to May 2022 (2310 of 5618 visits). Patients in the telemedicine-only group had fewer mean (SD) appointments per year (2.1 [0.8] appointments per 12 months) than those in the in-person and mixed follow-up groups (2.5 [1.1] appointments per 12 months and 2.9 [0.9] appointments per 12 months, respectively; rate ratio of appointments per 12 months comparing telemedicine to in-person, 0.803; 95% CI, 0.771-0.836; rate ratio comparing telemedicine to mixed follow-up groups, 0.699; 95% CI, 0.674-0.725; *P* < .001 for comparison of rate of appointments for in-person and mixed follow-up groups with telemedicine) (Table 1). Patients who used telemedicine only also had fewer follow-up HbA_1c_ measurements per 12 months than those who used in-person and mixed follow-up.

### Glycemic Outcomes by Care Modality

**Figure 2.**
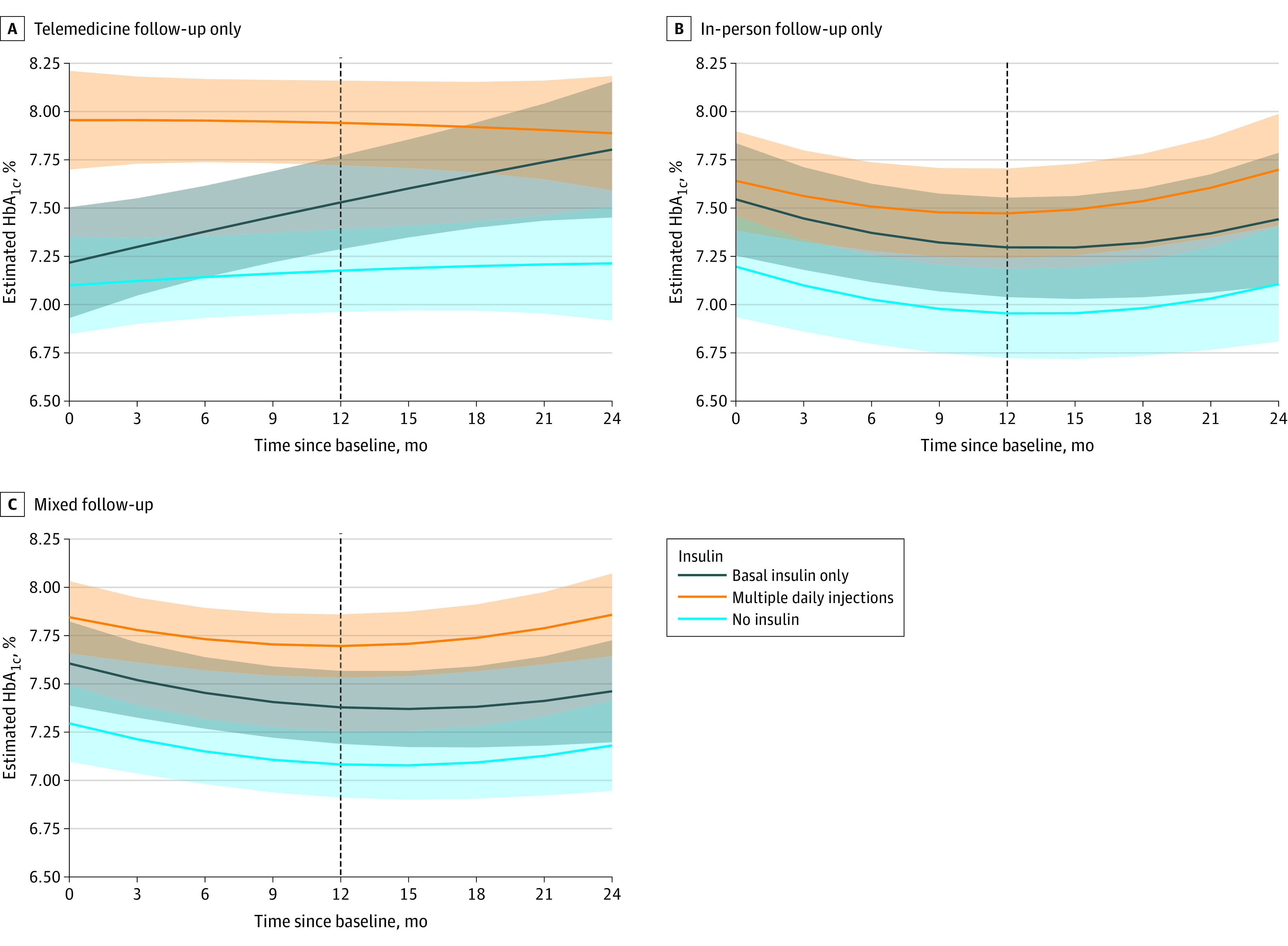
Adjusted Hemoglobin A_1c_ (HbA_1c_) Levels by Follow-Up Care Modality and Baseline Insulin Regimen Adjusted 12-month HbA_1c_ changes are model-based estimates derived from linear mixed modeling of repeated measures of HbA_1c_ adjusted for patient age, sex, race, ethnicity, social deprivation index, rurality, baseline HbA_1c_, and body mass index; patients were nested within practitioners. To convert to proportion of total hemoglobin, multiply by 0.01.

There was no significant change in HbA_1c_ from baseline to 12 months in the telemedicine-only group (−0.06%; 95% CI, −0.26 to 0.14), while the in-person and mixed follow-up groups demonstrated HbA_1c_ improvement (estimated change for in-person group, −0.37; 95% CI, −0.59 to −0.15; estimated change for mixed group, −0.22; 95% CI, −0.38 to −0.07) (Table 2). There was no significant change in the secondary outcome of HbA_1c_ change from baseline to 24 months across any care modality (Table 2). Model-derived trajectories of HbA_1c_ over time differed within care modalities based on insulin regimen (Figure 2). In the telemedicine group, there was minimal estimated HbA_1c_ change over time among patients not on insulin and those on MDI, while HbA_1c_ increased steadily from baseline to 24 months for patients on basal insulin. In contrast, among the in-person and mixed follow-up cohorts, adjusted HbA_1c_ declined from baseline to 12 months with subsequent increase from 12 to 24 months for patients in all 3 insulin groups.

**Table 2.** Adjusted HbA_1c_ Change From Baseline for Each Follow-Up Care Modality

Care modality	Primary outcome: adjusted 12-mo HbA_1c_ change from baseline (95% CI), percentage point^a^	*P* value	Secondary outcome: adjusted 24-mo HbA_1c_ change from baseline (95% CI), percentage point^a^	*P* value
Entire cohort (N = 3778)				
Telemedicine only (n = 1182)	−0.06 (−0.26 to 0.14)	.55	0.02 (−0.24 to 0.29)	.86
In-person only (n = 1049)	−0.37 (−0.59 to −0.15)	<.001	−0.19 (−0.46 to 0.07)	.15
Mixed follow-up (n = 1547)	−0.22 (−0.38 to −0.07)	.004	−0.11 (−0.31 to 0.09)	.27
Baseline HbA_1c _ ≥8% subgroup (n = 1198)				
Telemedicine only (n = 381)	−0.43 (−0.97 to 0.10)	.11	−0.32 (−1.00 to 0.36)	.35
In-person only (n = 280)	−1.80 (−2.44 to −1.15)	<.001	−1.81 (−2.57 to −1.04)	<.001
Mixed follow-up (n = 537)	−1.10 (−1.44 to −0.77)	<.001	−0.95 (−1.42 to −0.49)	<.001

Abbreviation: HbA_1c_, hemoglobin A_1c_.

SI Conversion: to convert to proportion of total hemoglobin, multiply by 0.01.

^a^ Adjusted 12-month HbA_1c_ changes are model-based estimates derived from linear mixed modeling of repeated measures of HbA_1c_ adjusted for patient age, sex, race, ethnicity, social deprivation index, rurality, baseline HbA_1c_, and body mass index; patients were nested within practitioners.

### Glycemic Outcomes by Clinical Complexity

In all 3 cohorts, patients prescribed basal insulin had worse adjusted HbA_1c_ changes at 12 months compared with those not prescribed insulin (Table 3). Similar trends were seen at 24 months, but the estimated difference in HbA_1c_ change between patients prescribed basal insulin and those not prescribed insulin was significant only for the telemedicine group. For patients prescribed MDI compared with those not prescribed insulin, estimated HbA_1c_ change was worse at both 12 and 24 months across all modalities (Table 3). In difference-in-difference analysis, HbA_1c_ change from baseline to 12 months for patients prescribed MDI vs no insulin was significantly worse in the telemedicine group than the in-person group (estimated difference in HbA_1c_ change, 0.25% higher; 95% CI, 0.02 to 0.47; *P* = .03) but was not significantly different between the mixed and telemedicine groups (estimated difference in HbA_1c_ change, 0.15% higher for telemedicine only; 95% CI, −0.36 to 0.05; *P* = .05). Similarly, outcomes of telemedicine vs in-person care among patient cohorts defined according to insulin use demonstrate significantly worse HbA_1c_ changes at 12 months for telemedicine vs in-person care among patients on MDI (estimated difference in HbA_1c_ change, −0.47%; 95% CI, −0.78 to −0.15) for in-person vs telemedicine only (eTable 3 in Supplement 1). There was no significant association of cardiovascular or psychological comorbidities with HbA_1c_ changes at 12 or 24 months in any care modality cohort (Table 3).

**Table 3.** Adjusted Difference in HbA_1c_ Change for Those With Insulin Use or Comorbidities vs Without for Each Follow-Up Care Modality

Clinical characteristic	Primary outcome: difference in adjusted HbA_1c_ change at 12 mo (95% CI), percentage point^a^	*P* value	Secondary outcome: difference in adjusted HbA_1c_ change at 24 mo (95% CI, percentage point)^a^	*P* value
Telemedicine only follow-up, entire cohort (n = 1182)				
Basal insulin vs no insulin	0.35 (0.15 to 0.56)	<.001	0.59 (0.22 to 0.96)	<.001
MDI vs no insulin	0.77 (0.59 to 0.94)	<.001	0.67 (0.37 to 0.98)	<.001
Cardiovascular vs none	0.04 (−0.12 to 0.19)	>.99	−0.01 (−0.27 to 0.26))	>.99
Psychological vs none	−0.001 (−0.15 to 0.15)	>.99	0.02 (−0.24 to 0.28	>.99
In-person only follow-up, entire cohort (n = 1049)				
Basal insulin vs no insulin	0.34 (0.13 to 0.55)	<.001	0.34 (−0.01 to 0.68)	.06
MDI vs no insulin	0.52 (0.34 to 0.70)	<.001	0.59 (0.30 to 0.88)	<.001
Cardiovascular vs none	−0.01 (−0.16 to 0.15)	.99	0.02 (−0.23 to 0.26)	>.99
Psychological vs none	0.05 (−0.11 to 0.21)	.99	0.04 (−0.23 to 0.31)	>.99
Mixed follow-up, entire cohort (n = 1547)				
Basal insulin vs no insulin	0.30 (0.12 to 0.47)	<.001	0.28 (−0.00 to 0.57)	.06
MDI vs no insulin	0.61 (0.46 to 0.76)	<.001	0.68 (0.43 to 0.92)	<.001
Cardiovascular vs none	0.01 (−0.12 to 0.14)	>.99	0.04 (−0.16 to 0.25)	>.99
Psychological vs none	−0.05 (−0.18 to 0.08)	.75	−0.04 (−0.25 to 0.17)	>.99
Telemedicine only follow-up, subgroup baseline HbA_1c_ ≥8% (n = 381)				
Basal insulin vs no insulin	0.44 (−0.10 to 0.99)	.14	0.68 (−0.30 to 1.65)	.24
MDI vs no insulin	0.95 (0.45 to 1.44)	<.001	0.67 (−0.17 to 1.51)	.15
Macrovascular vs none	−0.18 (−0.55 to 0.19)	.57	−0.37 (−1.0 to 0.27)	.39
Psychological vs none	0.31 (−0.05 to 0.68)	.11	0.39 (−0.22 to 1.01)	.31
In-person only follow-up, subgroup baseline HbA_1c_ ≥8% (n = 280)				
Basal insulin vs no insulin	0.42 (−0.27 to 1.11)	.34	0.09 (−1.05 to 1.23)	>.99
MDI vs no insulin	0.58 (−0.04 to 1.21)	.07	0.35 (−0.64 to 1.33)	.87
Macrovascular vs none	−0.06 (−0.48 to 0.36)	>.99	−0.28 (−0.95 to 0.40)	.72
Psychological vs none	0.09 (−0.36 to 0.55)	>.99	0.20 (−0.54 to 0.93)	>.99
Mixed follow-up, subgroup baseline HbA_1c_ ≥8% (n = 537)				
Basal insulin vs no insulin	0.50 (−0.03 to 1.04)	.07	0.28 (−0.61 to 1.16)	.96
MDI vs no insulin	0.79 (0.31 to 1.26)	<.001	0.52 (−0.22 to 1.26)	.23
Macrovascular vs none	0.13 (−0.17 to 0.43)	.64	0.26 (−0.22 to 0.73)	.46
Psychological vs none	−0.00 (−0.30 to 0.29)	>.99	0.12 (−0.37 to 0.60)	>.99

Abbreviations: HbA_1c_, hemoglobin A_1c_; MDI, multiple daily injections.

SI conversion factor: to convert to proportion of total hemoglobin, multiply by 0.01.

^a^ Adjusted differences in HbA_1c_ change are model-based estimates of the difference in HbA_1c_ change from baseline between each category compared with the reference group, as indicated. These model-based estimates were obtained from linear mixed modeling of repeated measures of HbA_1c_ adjusted for patient age, sex, race, ethnicity, social deprivation index, rurality, baseline HbA_1c_, and body mass index; patients were nested within practitioners.

### Subgroup Analysis: Baseline HbA_1c_ of 8% or Higher

Among patients whose baseline HbA_1c_ was 8% or higher, those who used telemedicine only had no significant change in adjusted HbA_1c_ at 12 or 24 months (Table 2). However, patients who used in-person or mixed follow-up had significant improvement in adjusted HbA_1c_ at both 12 and 24 months, mirroring patterns observed for the overall cohort. Among patients who used telemedicine or mixed follow-up, those prescribed MDI had a worse estimated change in HbA_1c_ at 12 months compared with those not prescribed insulin; there was no significant difference among patients who used in-person follow-up only (Table 3). The estimated difference in HbA_1c_ change at 24 months for patients prescribed either basal or MDI compared with no insulin was not significant across any care modality in this subgroup. As in the overall cohort, cardiovascular and psychological comorbidities were not significantly associated with glycemic outcomes in any care modality.

## Discussion

In this retrospective study of adults who received endocrinology care for T2D in a large health system from 2020 to 2022, patients accessing care through telemedicine alone had worse glycemic outcomes compared with patients who transitioned to in-person or mixed care. These findings build on and contrast with prior studies conducted in the primary care setting, which demonstrated similar glycemic outcomes of telemedicine and in-person care for T2D.^10,11,13,33^ Patients with T2D who receive endocrinology care and have more complex care needs, including those who use insulin or have HbA_1c_ above goal, may not be well served by telemedicine care alone as currently implemented.

Telemedicine emerged as a prominent modality of diabetes care delivery during the COVID-19 pandemic, but utilization patterns have changed over time. Our findings on patient subgroups who rely on telemedicine to access specialty diabetes care are consistent with prior work and identify new characteristics associated with ongoing telemedicine use. We found that younger,^34,35,36,37,38^ female,^10,35,36,37,38^ and urban-dwelling^39,40,41^ patients were more likely to use telemedicine only, similar to previous data in primary care and endocrinology settings. Black patients in our study were more likely to use telemedicine only, while prior evidence on the association of race and ethnicity with telemedicine use is mixed.^35,36,37,38,42,43^ We found that patients with less complex diabetes were more likely to use telemedicine only. In addition, our findings add new evidence that telemedicine may be particularly important for ensuring access to endocrinology care for patients who have psychological comorbidities, which are known to impact diabetes self-management and outcomes, and may require additional support to achieve treatment goals.^44,45^

In contrast to our findings, studies conducted earlier in the pandemic in primary care or general diabetes populations found similar glycemic control between patients receiving telemedicine and in-person diabetes care.^10,11,12,13,33^ There are several potential explanations for the observed lack of HbA_1c_ improvement among patients using telemedicine alone to access endocrinology care in our study. First, patient-level factors that may lead to preferential use of telemedicine care can also impact diabetes self-management and health care access in general. The telemedicine group was more urban dwelling, younger, more likely to be Black and female, and may face unmeasured competing demands to diabetes self-management, such as caregiving responsibilities, transportation barriers, or work schedules.^46^ Telemedicine-only users also had lower care utilization, including less frequent appointments and HbA_1c_ testing, which may lead to more clinical inertia^47^ and less intensification of treatment by endocrinology practitioners. Although it is not clear whether lower care utilization was driven by patients, practitioners, or systemic barriers, prior studies of diabetes telemedicine have found similar results.^13,48,49^ Additionally, differences in patient-practitioner communication via telemedicine, including difficulty building rapport,^50,51^ may lead to differences in both patient self-management and practitioner treatment decisions.

Another potential explanation for inferior glycemic outcomes in the telemedicine group is that strategies to support glycemic improvement that are available during in-person appointments have not consistently been translated to telemedicine care. Care elements which may be particularly influential for patients with elevated HbA_1c_ or complex insulin regimens, such as self-management education and support, sharing of home blood glucose data through device downloads or written logs, and educational resources for initiation of diabetes technology or new medications, may not currently be routinely delivered through telemedicine or available at the point-of-care during telemedicine visits. In our prior work in this care setting, practitioners described how inferior availability of glucose data limited their ability to intensify treatment through telemedicine.^19^ Implementation of approaches to overcome these differences, such as team-based virtual care and technological tools to automate blood glucose data sharing, are needed to ensure all patients receive high-quality diabetes care regardless of care modality.^52^

### Strengths and Limitations

There are a number of strengths and limitations to this study. This is the first study, to our knowledge, to examine outcomes of telemedicine care specifically in the endocrinology setting and according to clinical factors that impact treatment complexity. Although demographic variables that differed between groups were included as covariates, cohorts were not balanced on potentially confounding baseline characteristics. Factors including treatment complexity and glycemic control, as well as geographic and transportation barriers, may have impacted whether patients received care via telemedicine or in-person care; thus, findings reflect glycemic outcomes for clinical patients who received care via each modality, and do not indicate causal associations. This study was performed in a single health system, and patients had to use at least 1 medication for diabetes and were predominantly white and urban; thus, results may not generalize to other settings with different infrastructure for telemedicine care, rural areas, or patient populations with more racial and ethnic diversity or who have diet-controlled diabetes. In addition, HbA_1c_ values were not consistently captured for patients who had testing done at facilities that do not communicate with the electronic medical records. However, demographic variables which differed between included patients and those excluded due to lack of follow-up HbA_1c_ value (11.6% of eligible patients) were controlled for in models, limiting the impact of this issue on our results. Finally, with loss to follow-up over time, there were fewer patients who had HbA_1c_ results at 24 months compared with 12 months; thus, these results should be interpreted as exploratory only.

## Conclusions

In this cohort study of adults who received endocrinology specialist care for T2D in a large health system from 2020 to 2022, patients who accessed care through telemedicine alone had worse glycemic outcomes compared with patients who transitioned to in-person or mixed care. Since some patients with barriers to in-person endocrinology care will continue to rely on telemedicine to access care, structured approaches to ensure routine delivery of high-quality team-based diabetes care are needed. Translation of successful strategies from clinical trials into routine telemedicine care, especially targeted toward adults with more complex diabetes, is critical to improve clinical outcomes for patients who rely on this care modality.

## Notes

Supplement 1. **eTable 1. ***ICD-10* Codes Used to Identify Comorbid Conditions **eTable 2. **Characteristics of Modeled vs Excluded Patients According to Presence of Follow-Up HbA_1c_ **eTable 3. **Supplemental Analysis: Adjusted Difference HbA_1c_ Change for Each Care Modality Cohort by Insulin Use

Supplement 2.
Data Sharing Statement
