# Supplementary material for: Patterns of Telemedicine Use and Glycemic Outcomes of Endocrinology Care for Patients With Type 2 Diabetes
Source: JAMA Netw Open. 2023 Dec 6;6(12):e2346305. doi: 10.1001/jamanetworkopen.2023.46305 (PMC10701613; doi:10.1001/jamanetworkopen.2023.46305)
Supplement: Supplement 1. — eTable 1. ICD-10 Codes Used to Identify Comorbid Conditions eTable 2. Characteristics of Modeled vs Excluded Patients According to Presence of Follow-Up HbA1c eTable 3. Supplemental Analysis: Adjusted Difference HbA1c Change for Each Care Modality Cohort by Insulin Use [file jamanetwopen-e2346305-s001.pdf]

Supplemental Online Content

Zupa MF, Vimalananda VG, Rothenberger SD, et al. Patterns of telemedicine use and glycemic outcomes of endocrinology care for type 2 diabetes. *JAMA Netw Open*. 2023;6(12):e2346305. doi:10.1001/jamanetworkopen.2023.46305

eTable 1. *ICD-10* Codes Used to Identify Comorbid Conditions
eTable 2. Characteristics of Modeled vs Excluded Patients According to Presence of Follow-Up HbA_{1c}
eTable 3. Supplemental Analysis: Adjusted Difference HbA1c Change for Each Care Modality Cohort by Insulin Use

This supplemental material has been provided by the authors to give readers additional information about their work.

© 2023 Zupa MF et al. *JAMA Network Open.*

eTable 1. ICD-10 Codes Used to Identify Comorbid Conditions[a]

Cardiovascular	
Coronary artery disease	I20.X, I21.X, I22.X, I23.X, I24.X, I25.X
Congestive heart failure	I50.X, I42.X, I110.X, J19.X
Arrhythmia	I47.X, I48.X, I49.X
Cerebrovascular disease	I60.X, I61.X, I62.X, I63.X, I64.X, I65.X, I66.X, I67.X, I68.X, I69.X, DG45.X, DG46.X
Vascular disease	I70.X, I71.X, I72.X, I73.X, I74.X, I75.X, I76.X, I77.X, I78.X, I79.X
Gangrene	I96.X, E11.5X
Psychologic	
Psychotic disorders	F20.X, F21.X, F22.X, F23.X, F24.X, F25.X, F28.X, F29.X
Mood disorders	F30.X, F31.X, F32.X, F33.X, F34.X, F39.X
Post Traumatic Stress Disorder	F43.X
Anxiety disorders	F40.X, F41.X
Alcohol use disorder	F10.X
Substance use disorder	F11.X, F12.X, F13.X, F14.X, F15.X, F16.X, F18.X, F19.X

a. Based on prior work by Pentakota et al[25]

© 2023 Zupa MF et al. *JAMA Network Open.*

eTable 2. Characteristics of Modeled vs Excluded Patients According to Presence of Follow-Up HbA_{1c}

Characteristic	Total (N=4,275)	Included (N=3,778)	Excluded (N=497)	P-Value[a]
Age (years); Mean (SD)	59.4 (13.3)	60.3 (12.7)	52.8 (15.8)	<0.001
Gender				<0.001
Female N (%)	2,547 (60%)	2,201 (58%)	346 (70%)	
Male N (%)	1,728 (40%)	1,577 (42%)	151 (30%)	
Race N (%)				0.02
White	3,759 (88%)	3,332 (88%)	427 (86%)	
Black	347 (8%)	300 (8%)	47 (9%)	
Asian	87 (2%)	81 (2%)	6 (1%)	
Other/Missing	82 (2%)	65 (1%)	17 (3%)	
Hispanic or Latino N (%)	78 (2%)	66 (2%)	12 (2%)	<0.001
Not specified/missing	197 (5%)	158 (4%)	39 (8%)	
SDI Score; Mean (SD)	40.7 (24.0)	40.5 (23.9)	41.5 (25.0)	0.40
RUCA; N (%)				0.02
Urban	3,024 (71%)	2,645 (70%)	379 (76%)	
Suburban	921 (22%)	836 (22%)	85 (17%)	
Rural	330 (8%)	297 (8%)	33 (7%)	
HbA1c (%); Mean (SD)	7.5 (1.8)	7.6 (1.7)	7.2 (2.1)	<0.001
Insulin N (%)				<0.001
No insulin	1,763 (41%)	1,476 (39%)	287 (58%)	
Basal only	703 (16%)	652 (17%)	51 (10%)	
MDI	1,809 (42%)	1,650 (44%)	159 (32%)	
Non-insulin med count; Mean (SD)	1.8 (1.1)	1.9 (1.1)	1.5 (1.0)	<0.001
BMI categorized; N (%)				<0.001
18.5-24.9	217 (5%)	196 (5%)	21 (4%)	
25-29.9	693 (16%)	639 (17%)	54 (11%)	
30-34.9	965 (23%)	880 (23%)	85 (17%)	
35-39.9	757 (18%)	688 (18%)	69 (14%)	
≥40	867 (20%)	770 (20%)	97 (20%)	
Missing	776 (18%)	605 (16%)	171 (34%)	
Comorbid conditions N (%)				
Macrovascular	1,526 (36%)	1,393 (37%)	133 (27%)	<0.001
Psychologic	1,413 (33%)	1,246 (33%)	167 (34%)	0.78
Appointments/12 months; Mean (SD)	2.5 (1.0)	2.6 (1.0)	1.7 (0.7)	<0.001
Number of follow-up HbA1c test results/12 months; Mean (SD)	1.5 (0.9)	1.7 (0.8)	0 (0.0)	<0.001

a. For continuous variables, Kruskal-Wallis test was used; for categorical variables, Chi-square test was used

© 2023 Zupa MF et al. *JAMA Network Open.*

eTable 3. Supplemental Analysis: Adjusted Difference HbA1c Change for Each Care Modality Cohort by Insulin Use

**Clinical Characteristic**	**Difference in Estimated HbA1c change at 12-months**[a] (95% CI)	**P value**	**Difference in Estimated HbA1c change at 24-months**[a] (95% CI)	**P-value**
**No insulin (n=1,476)**				
Mixed vs. telemedicine only	-0.09 (-0.36, 0.17)	0.990	-0.03 (-0.41, 0.34)	>0.999
Office vs telemedicine only	-0.22 (-0.53, 0.09)	0.324	-0.11 (-0.52, 0.31)	>0.999
**Basal insulin only (n=652)**				
Mixed vs. telemedicine only	-0.15 (-0.45, 0.15)	0.652	-0.34 (-0.78, 0.09)	0.250
Office vs telemedicine only	-0.23 (-0.58, 0.12)	0.382	-0.36 (-0.85, 0.13)	0.297
**Multiple daily injections (n=1,650)**				
Mixed vs. telemedicine only	-0.24 (-0.51, 0.02)	0.146	-0.03 (-0.39, 0.33)	>0.999
Office vs telemedicine only	-0.47 (-0.78, -0.15)	0.007	-0.19 (-0.60, 0.22)	0.734

a. Adjusted differences in HbA1c change are model-based estimates of the difference in HbA1c change from baseline between each category compared to the reference group, as indicated. These model-based estimates were obtained from linear mixed modeling of repeated measures of HbA1c adjusted for patient age, gender, race, ethnicity, social deprivation index, rurality, baseline HbA1c, and BMI; patients were nested within providers

© 2023 Zupa MF et al. *JAMA Network Open.*
