# Supplementary material for: Patterns of Telemedicine Use and Glycemic Outcomes of Endocrinology Care for Patients With Type 2 Diabetes
Source: JAMA Netw Open. 2023 Dec 6;6(12):e2346305. doi: 10.1001/jamanetworkopen.2023.46305 (PMC10701613; doi:10.1001/jamanetworkopen.2023.46305)
Supplement: Supplement 2. — Data Sharing Statement [file jamanetwopen-e2346305-s002.pdf]

Data Sharing Statement

Zupa. Patterns of Telemedicine Use and Glycemic Outcomes of Endocrinology Care for Patients With Type 2 Diabetes. *JAMA Netw Open*. Published December 06, 2023. doi:10.1001/jamanetworkopen.2023.46305

Data
Data available: No

Additional Information
Explanation for why data not available: The data used in this study include potentially identifying patient information including dates and zip codes, and thus are not available in their entirety. A limited dataset is available from the authors upon reasonable request and with permission of relevant institutional review boards.
